# The Modulation of Gamma Oscillations by Methamphetamine in Rat Hippocampal Slices

**DOI:** 10.3389/fncel.2019.00277

**Published:** 2019-06-21

**Authors:** Yanan Li, Xin’e Xie, Hang Xing, Xiang Yuan, Yuan Wang, Yikai Jin, Jiangang Wang, Martin Vreugdenhil, Ying Zhao, Ruiling Zhang, Chengbiao Lu

**Affiliations:** ^1^The Second Affiliated Hospital, Xinxiang Medical University, Xinxiang, China; ^2^Key Laboratory for the Brain Research of Henan Province, Department of Physiology, Xinxiang Medical University, Xinxiang, China; ^3^Department of Neurology, Henan Provincial People’s Hospital, Zhengzhou, China; ^4^The First Affiliated Hospital, College of Clinical Medicine, Henan University of Science and Technology, Luoyang, China; ^5^Department of Health Sciences, Birmingham City University, Birmingham, United Kingdom; ^6^Key Laboratory of Clinical Psychopharmacology, School of Pharmacy, Xinxiang Medical University, Xinxiang, China

**Keywords:** methamphetamine, γ oscillation, hippocampus, Akt/PKB, DR, NMDAR

## Abstract

Gamma frequency oscillations (γ, 30–100 Hz) have been suggested to underlie various cognitive and motor functions. The psychotomimetic drug methamphetamine (MA) enhances brain γ oscillations associated with changes in psychomotor state. Little is known about the cellular mechanisms of MA modulation on γ oscillations. We explored the effects of multiple intracellular kinases on MA modulation of γ induced by kainate in area CA3 of rat ventral hippocampal slices. We found that dopamine receptor type 1 and 2 (DR1 and DR2) antagonists, the serine/threonine kinase PKB/Akt inhibitor and *N*-methyl-D-aspartate receptor (NMDAR) antagonists prevented the enhancing effect of MA on γ oscillations, whereas none of them affected baseline γ strength. Protein kinase A, phosphoinositide 3-kinase and extracellular signal-related kinases inhibitors had no effect on MA. We propose that the DR1/DR2-Akt-NMDAR pathway plays a critical role for the MA enhancement of γ oscillations. Our study provides an new insight into the mechanisms of acute MA on MA-induced psychosis.

## Introduction

Neuronal synchronization at γ frequency band (30–100 Hz, γ oscillations) emerges from activated neuronal networks, consisting of mutually connected principal cells and inhibitory cells (Fisahn et al., 1998). γ oscillations provide a millisecond-precision timing matrix that facilitates inter-neuronal communication and information transfer (Akam and Kullmann, 2014). In addition to synchronizing neuronal activity locally, oscillatory activity contributes to inter-region or long-range synchronization (Konig et al., 1995, Colgin, 2011, Fries, 2015). γ oscillations are implicated in a variety of cognitive functions, including attention, perceptual binding and memory (Womelsdorf et al., 2007, Buzsaki and Wang, 2012). Interestingly, γ oscillations have been associated with lucid dreaming and emotion processes (Garcia-Garcia et al., 2010, Scarpelli et al., 2019).

Aberrant γ oscillations have been considered as a biomarker of a variety of neurological and psychiatric disorders, including Alzheimer’s disease (Mably and Colgin, 2018), ADHD (Barry and Clarke, 2013), schizophrenia and major depression (Yener and Basar, 2013, Fitzgerald and Watson, 2018). Enhanced γ oscillation power has been associated with psychosis. γ oscillation abnormalities in schizophrenia include abnormal increases in baseline power as well as deficits in evoked oscillations (Hirano et al., 2015). The psychotomimetic drug methamphetamine (MA) causes psychosis in users that resemble positive symptoms of schizophrenia (Wearne and Cornish, 2018). MA enhances γ oscillations in the nucleus accumbens and causes psychosis-like repetitive behaviors in rodents (Morra et al., 2012). Amphetamine, a metabolite of MA, enhances γ oscillations in the neocortex (Pinault, 2008; Qi et al., 2018) and hippocampus (Leung and Ma, 2018). Whereas hippocampal γ oscillations are crucially involved in a triangular relation with the nucleus accumbens and prefrontal cortex (Ma and Leung, 2010), the effect of MA on hippocampal γ oscillations is yet unknown.

γ oscillations can be reliably induced in the hippocampus *in vitro* by the glutamate receptor agonist kainate (KA) (Cunningham et al., 2003; Ainsworth et al., 2011; Lu et al., 2012; Balleza-Tapia et al., 2018). Although at higher concentration KA can cause epileptiform activity in the hippocampus (Fisher and Alger, 1984), at low concentration KA reliably induces γ oscillations (Hajos et al., 2000), which show a high degree of similarity with γ oscillations *in vivo* (Butler and Paulsen, 2015), including the crucial role of fast-spiking perisomatic parvalbumin-containing interneurons (Fisahn et al., 2004; Mann and Paulsen, 2005; Bartos et al., 2007; Ainsworth et al., 2011; Buzsaki and Wang, 2012). Furthermore*, in vitro* hippocampal γ power can serve as an index of *in vivo* CA3 γ strength (Lu et al., 2011). Whereas *in vivo*, hippocampal γ oscillation power is modulated by the theta rhythm, driven by the medial septum, and by other midbrain influences, KA-induced γ oscillations in acute hippocampal slices provide a model to study γ generating network in isolation, which allows for the study of the effect of MA on cellular and molecular properties in the γ generating network.

Several pathways affected by MA may be implicated in the modulation of hippocampal γ oscillations. MA increases extracellular dopamine levels both by inhibiting the dopamine transporter and by increasing dopamine release (Ares-Santos et al., 2013), probably through activation of trace amine-associated receptor 1, which inhibits uptake and promoting efflux by monoamine transporters (Miller, 2011). MA affects dopamine-mediated intracellular signaling pathways (Sulzer, 2011), like the classical cAMP-protein kinase A (PKA) pathway, which is activated by DR1 activation and inhibited by DR2 activation. Other pathways involved include phosphoinositide 3-kinase (PI3K)-dependent or independent Akt signaling (Beaulieu et al., 2007a) and extracellular signal-regulated kinase (ERK), which is activated by both DR1 and DR2 agonists (Brami-Cherrier et al., 2002; Beaulieu et al., 2011).

Since acute application of MA increases brain glutamate concentration, GluN2B-mediated synaptic currents (Li et al., 2017) and NR1 expression (Kerdsan et al., 2009), altered NMDAR-mediated pathways may modulate γ oscillations, as previously shown by nicotine (Wang et al., 2015) or ethanol (Wang et al., 2016).

In this study, we test the role of acute MA on hippocampal γ oscillations *in vitro* and what signaling pathways are involved in MA modulation of γ. We found that MA caused a robust enhancement of hippocampal γ oscillations, through the activation of DR-Akt-NMDAR signaling pathways.

## Materials and Methods

### Animals

All animal use procedures were approved by the Ethics Committees at the Xinxiang Medical University for the Care and Use of Laboratory Animals, and all efforts were made to minimize animal suffering and the number of animals used. Ventral hippocampal slices were prepared from Sprague Dawley rats (male, 4–5 week-old). The animals were anesthetized by intraperitoneal injection of Sagatal (sodium pentobarbitone, 100 mg kg^–1^, Rhône Mérieux Ltd., Harlow, United Kingdom). When all pedal reflexes were abolished, the animals were perfused intracardially with chilled (5°C), oxygenated artificial cerebrospinal fluid (ACSF) in which the sodium chloride had been replaced by iso-osmotic sucrose. This sucrose-ACSF contained (in mM): 225 sucrose, 3 KCl, 1.25 NaH_2_PO_4_, 24 NaHCO_3_, 6 MgSO_4_, 0.5 CaCl_2_, and 10 glucose (pH: 7.4). Horizontal slices (400 μm) of rat brain were cut at 5°C in the sucrose-ACSF, using a Leica VT1000S vibratome (Leica Microsystems, Milton Keynes, United Kingdom) and stored at room temperature at the interface between recording ACSF and humidified carbogen (95% O_2_–5% CO_2_) until transferred to the recording chamber. The recording ACSF contained (in mM): 126 NaCl, 3 KCl, 1.25 NaH_2_PO_4_, 24 NaHCO_3_, 2 MgSO_4_, 2 CaCl_2_, and 10 Glucose (pH: 7.4).

### Electrophysiological Recording, Data Acquisition and Analysis

In the Haas type recording chamber, hippocampal slices were maintained at a temperature of 32°C at the interface between ACSF and warm humidified carbogen and were allowed to equilibrate in this medium for 1 h prior to recording. Extracellular field potentials were recorded from stratum pyramidale of CA3c, using glass microelectrodes containing ACSF (resistance 2–5 MΩ). Field potentials were amplified by Neurolog NL106 AC/DC amplifiers (Digitimer Ltd., Welwyn Garden City, United Kingdom) and band-pass filtered between 0.5 Hz and 2 kHz using Neurolog NL125 filters (Digitimer). Electromagnetic interference from the mains supply was eliminated from the recordings with the use of Humbug 50 Hz noise eliminators (Digitimer Ltd.). The recordings were digitized at a sample rate of 5–10 kHz using a CED 1401 plus ADC board (Cambridge Electronic Design, Cambridge, United Kingdom).

Data were analyzed off-line using Spike-2 software (Cambridge Electronic Design). Power spectra were generated to provide a quantitative measure of the frequency components. Power spectra were constructed for 60 s epochs using a fast Fourier transform algorithm. The area under the curve between 20 and 60 Hz was used to quantify the γ power.

All statistical tests were performed using SigmaStat software (Sysstat software, San Jose, CA, United States). Since data sets were not different from normal distributions, results are expressed as mean ± standard error of mean. Comparisons within slices were made with paired Student’s *t*-test. Comparisons between the effect of a drug and changes in time-matched controls were made with unpaired Student’s *t*-test. The effect of MA concentrations was tested by one-way ANAOVA.

Effects were considered statistically significant, if *P* < 0.05.

### Drugs

Methamphetamine was purchased from the National Institute for Food and Drug Control (Beijing, China). The selective DR1 antagonist SCH23390, DR2 antagonist raclopride, the mitogen-activated protein kinase inhibitor U0126 and the PI3K inhibitor wortmannin were purchased from Tocris Cookson Ltd. (Bristol, United Kingdom). The Akt/PKB inhibitor triciribine PKA inhibitor H89, NMDAR antagonist D-AP5 and the kainate receptor agonist kainate (KA) were obtained from Sigma-Aldrich (United Kingdom).

Stock solutions, at thousand times the final concentration, were made up in water or in DMSO, and stored in individual aliquots at −20°C. Final solutions were prepared freshly on the day of the experiment.

## Results

### MA Increases Kainate-Induced γ Oscillations

To investigate the effect of MA on the γ oscillation-generating network in area CA3, without modulatory influences of other brain areas, γ oscillations were induced in the hippocampal CA3 *in vitro*. The CA3 network was activated by perfusing hippocampal slices with 200 nM KA (Hajos et al., 2000).

**FIGURE 1:**
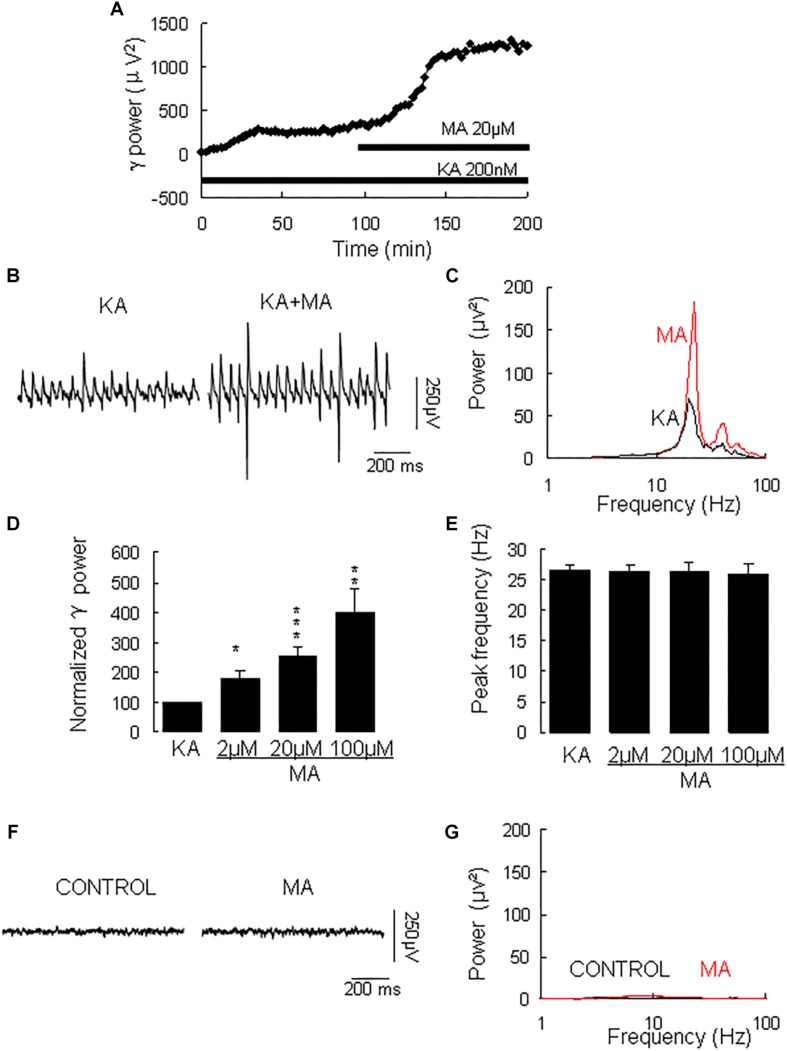
The influence of MA on KA-induced γ oscillations. **(A)** Typical example of the development of γ power in hippocampal area CA3, as function of time after the start of KA (200 μM). After γ power stabilized, MA (20 μM) was added. **(B)** Example of γ oscillations recorded in area CA3, induced by KA and after addition of MA. **(C)** Power spectra of the oscillatory activity in KA alone (black line) and after MA application (red line) for the slice in **B**. **(D)** The γ power (normalized to the KA only baseline value for continued KA only application (control) and for MA application at various concentrations (^*^P < 0.05; ^∗∗^*P* < 0.01; ^∗∗∗^*P* < 0.001). **(E)** Peak frequency of γ oscillations before and after MA application at various concentrations (details as in **D**). **(F)** Example of activity recorded in control aCSF and after application of MA (20 μM). **(G)** Power spectra of the oscillatory activity in control (black line) and after MA application (red line) for the slice in **F**.

γ oscillation amplitude reached a steady state usually after 40–120 min application of KA (example in Figure 1A, with a γ power of 1586 ± 528 μV^2^ (*n* = 30) and a peak frequency of 26.6 ± 1.0 Hz (*n* = 30, example in Figures 1B,C). MA was then added to the ACSF (2 μM, 20 μM, or 100 μM) and perfused for 60 min. MA concentration-dependently increased γ power by 79 ± 27% [*t*_(6)_ = −2.94, *P* = 0.026] for 2 μM, by 152 ± 32% [*t*_(14)_ = −2.94, *P* = 0.001, example in Figures 1B,C] for 20 μM and by 300 ± 77% [*t*_(10)_ = −3.86, *P* = 0.003] for 100 μM MA. The MA-induced increase in γ power was concentration-dependent [*F*_(2,30)_ = 3.977, *P* = 0.0132, Figure 1D]. MA did not affect the peak frequency of γ oscillations [*F*_(3,39)_ = 0.32, *P* = 0.815, Figure 1E]. To exclude the possibility that MA causes γ oscillations in addition to KA-induced γ oscillations, we tested the effect of MA in the absence of KA. Perfusion of MA alone (20 μM) for 60 min had no effect on baseline oscillatory activity (γ power in MA was 35.3 ± 9.4 μV^2^ vs. baseline 30.5 ± 10.2 μV^2^, *t*_(3)_ = −0.274, *P* = 0.802, example in Figures 1F,G). These results indicate that MA strongly facilitates KA-induced γ oscillations.

### The Effect of DR Antagonists on the MA-Mediated Enhancement of γ Oscillations

**FIGURE 2:**
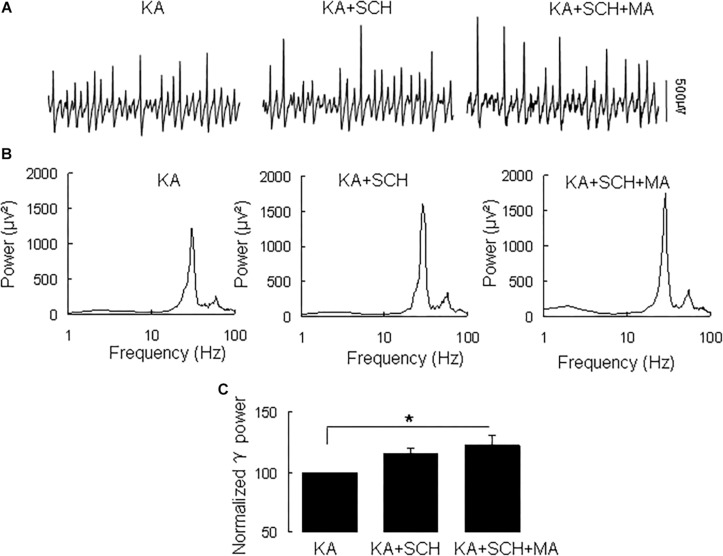
SCH23390 blocked the MA-induced increase in γ oscillations. **(A)** Recordings from CA3 in the presence KA alone, KA + 10 μM SCH23390 (SCH) and KA + 10 μM SCH23390 + 20 μM MA. **(B)** The power spectra of recordings in **A**. **(C)** γ power as percentage of preceding baseline for KA alone, KA + SCH23390 and KA + SCH23390 + MA (^*^P < 0.05).

Methamphetamine increases excitatory synaptic transmission in the hippocampus slice via activation of dopamine receptors (Swant et al., 2010). To determine whether and which dopamine receptor was involved in the MA-mediated enhancement of γ oscillations, we tested the effect of the dopamine receptor antagonists. In slices with stable γ oscillations, the DR1 antagonist SCH23390 (5 μM) was applied for 20 min, after which MA (20 μM) was added for 60 min. After 20 min in SCH23390 γ power was 116 ± 4% of baseline, not different from the control [*t*_(8)_ = −1.929, *P* = 0.090, Figure 2]. Addition of MA caused no significant change in γ power (105 ± 7% of SCH23390, *t*_(8)_ = −0.660, *P* = 0.528, Figure 2). These results indicate that the DR1 antagonist largely blocked the effect of MA on γ oscillations.

**FIGURE 3:**
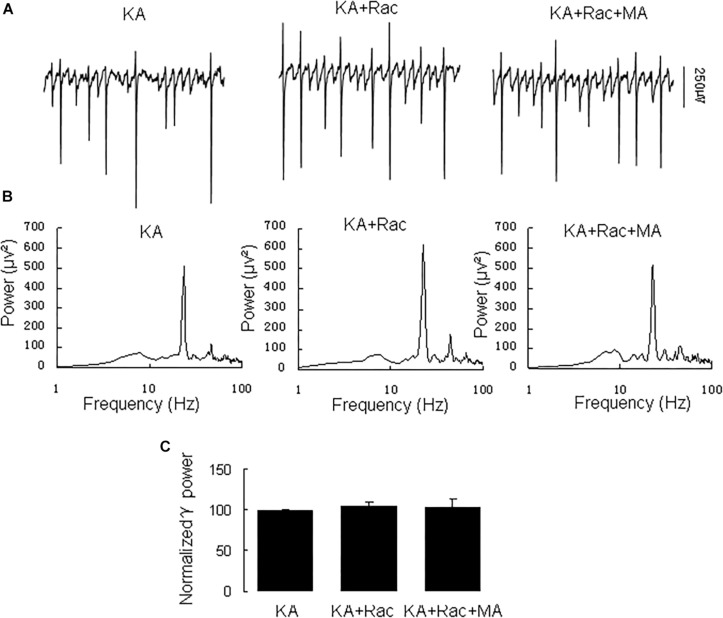
Raclopride blocked the MA-induced increase in γ oscillations. **(A)** CA3 recordings for KA alone, KA+ 10 μM Raclopride and KA+10 μM Raclopride + 20 μM MA. **(B)** The power spectra of recordings in **A**. **(C)** γ power as percentage of preceding baseline for KA alone, KA + Raclopride and KA+ Raclopride + MA.

Similarly, the DR2 antagonist raclopride prevented the γ oscillation-enhancing effect of MA. After 20 min in raclopride (10 μM) γ power was 105 ± 5% of baseline, not different from control [*t*_(8)_ = −1.268, *P* = 0.240, Figure 3]. Addition of MA (20 μM) did not enhance γ power [98 ± 5% of raclopide, *t*_(8)_ = 0.18, *P* = 0.861, Figure 3]. These results indicate that the DR2 antagonist blocked the effect of MA on γ oscillations. Therefore, our data suggest that both DR1 and DR2 are necessary for the MA-mediated enhancement of γ oscillations.

### The Effect of PKA Inhibition on the MA-Mediated Enhancement of γ Oscillations

**FIGURE 4:**
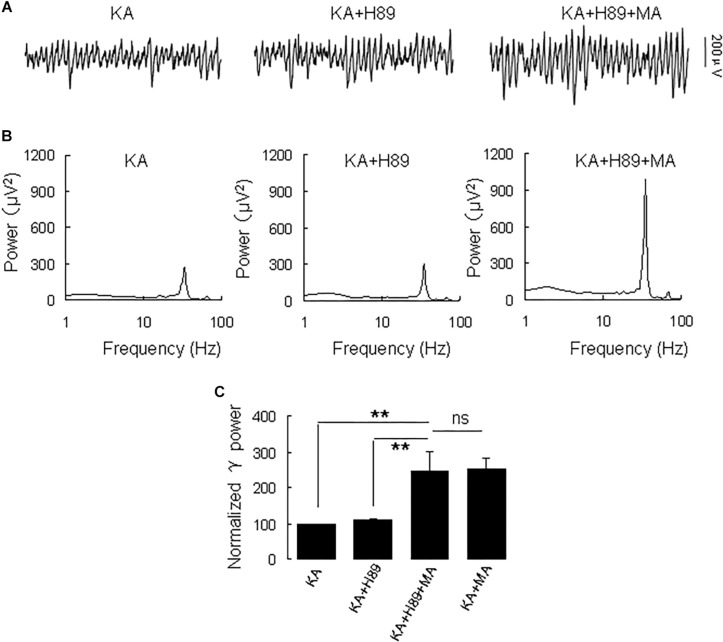
H89 does not affect the MA-induced increase in γ oscillations. **(A)** CA3 recordings for KA alone, KA + 10 μM H89 and KA + 10 μM H89 + 20 μM MA. **(B)** The power spectra of the recordings in **A**. **(C)** γ power as percentage of preceding baseline for KA alone, KA + H89, KA + H89 + MA and KA + MA (^*^P < 0.05, ^∗∗^*P* < 0.01, ns: not significant).

Because DR1 and DR2 mediate PKA activity in opposite ways, we tested the PKA inhibitor H89 on the MA-mediated enhancement of γ oscillations. Application of H89 (10 μM) alone had no effect on γ power (110 ± 4% of baseline, Figure 4), not different from control [*t*_(9)_ = −1.662, *P* = 0.131]. Further application MA (20 μM) increased γ power by 120 ± 44% of H89 [*t*_(9)_ = −2.647, *P* = 0.027, Figure 4], not different from the effect of MA alone [*t*_(23)_ = −0.092, *P* = 0.927]. These results indicate that PKA was not involved in the MA-induced effect on γ oscillations.

### The Effect of ERK Inhibition on the MA-Mediated Enhancement of γ Oscillations

**FIGURE 5:**
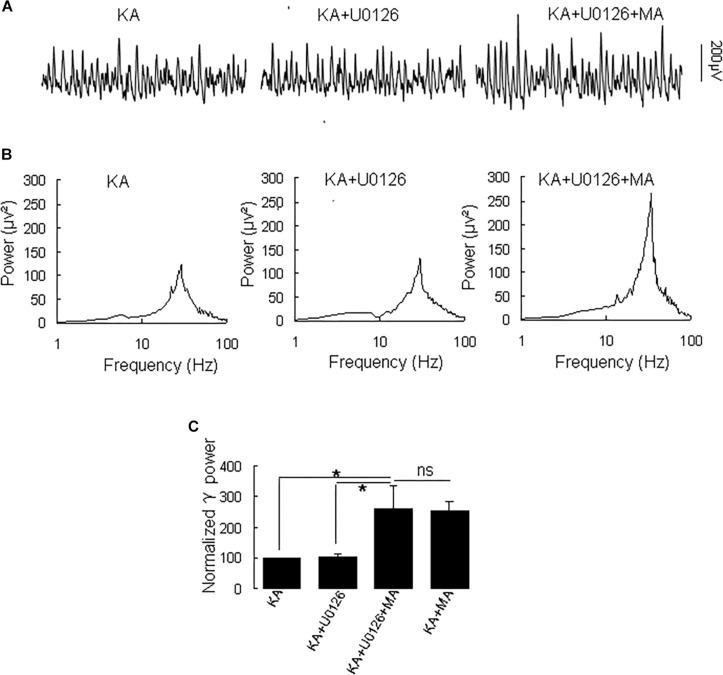
U0126 does not affect the MA-induced increase in γ oscillations. **(A)** CA3 recordings for KA alone, KA + 2.5 μM U0126 and KA + 2.5 μM U0126 + 20 μM MA. **(B)** The power spectra for the recordings in **A**. **(C)** γ power as percentage of preceding baseline for KA alone, KA+ U0126, KA+ U0126 + MA and KA+MA (^*^P < 0.05, ns: not significant).

Because MA induces ERK phosphorylation in the hippocampus (Cao et al., 2013) and ERK phosphorylation has been implicated in psychosis (Pereira et al., 2014), we tested the effect of the selective ERK inhibitor U0126 on the MA-mediated enhancement of γ oscillations. U0126 (2.5 μM) did not affect γ power [105 ± 6% of baseline, *t*_(5)_ = −0.474, *P* = 0.660, Figure 5]. Further application of MA (20 μM) increased γ power by 152 ± 56% from U0126 [*t*_(5)_ = −2.778, *P* = 0.039, Figure 5], not different from the increase by MA alone [*t*_(19)_ = −0.161, *P* = 0.874]. The results show that the ERK was not involved in MA-mediated enhancement of γ oscillations.

### The Effect of PI3K Inhibition on the MA-Mediated Enhancement of γ Oscillations

**FIGURE 6:**
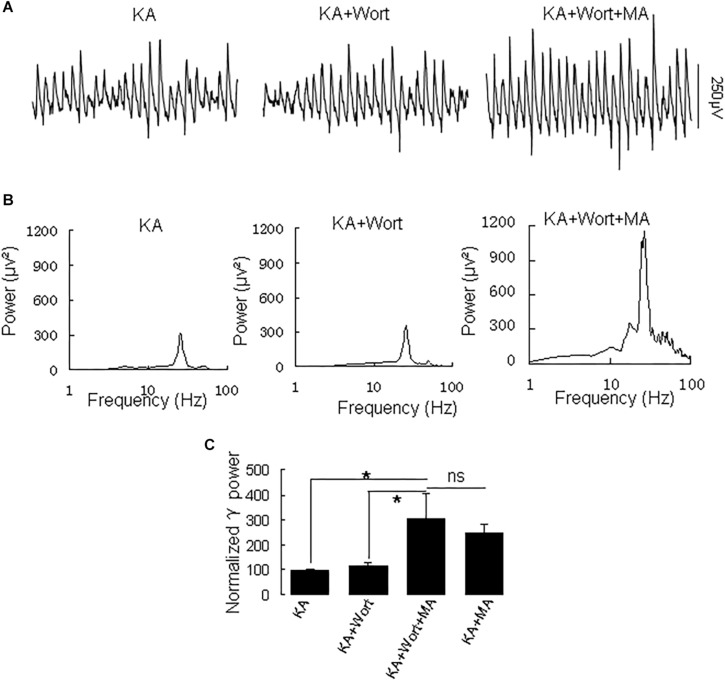
Wortmannin does not affect the MA-induced increase in γ oscillations. **(A)** CA3 recordings for KA alone, KA + 0.2 μM wortmannin (Wort) and KA + 0.2 μM wortmannin + 20 μM MA. **(B)** The power spectra for the recordings in **A**. **(C)** γ power as percentage of preceding baseline for KA alone, KA+ Wortmannin, KA + Wortmannin + MA and KA + MA (^*^P < 0.05, ns: not significant).

Because PI3K is implicated in the effects of the MA analog phentermine (Hong et al., 2016), we tested the effect of the PI3K inhibitor wortmannin on the effect of MA on γ oscillations. Application of wortmannin (200 nM) alone slightly increased γ power (to 119 ± 10% of baseline, not different from the control [*t*_(7)_ = −1.359, *P* = 0.223]. Further application of MA (20 μM) increased γ power by 137 ± 40% from wortmannin [*t*_(7)_ = −2.790, *P* = 0.027, Figure 6], not different from the enhancement by MA alone [*t*_(21)_ = −0.800, *P* = 0.432]. These results indicate that PI3K was not involved in MA-mediated enhancement of γ.

### Effect of Akt Inhibition on the MA-Mediated Enhancement of γ Oscillations

Because MA activates Akt (Nohesara et al., 2016), we tested the effect of the Akt inhibitor triciribine on the MA-mediated enhancement of γ oscillations.

**FIGURE 7:**
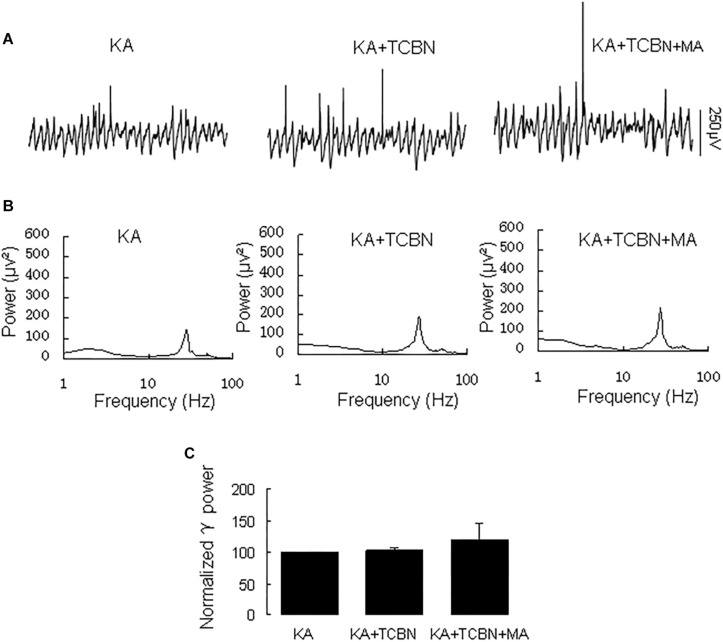
Triciribine blocked the MA-induced increase in γ oscillations. **(A)** CA3 recordings for KA alone, KA+ 5 μM triciribine and KA+5 μM triciribine +20 μM MA. **(B)** The power spectra for the recordings in **A**. **(C)** γ power as percentage of preceding baseline for KA, KA + triciribine and KA + triciribine + MA and KA + MA.

Application of triciribine (5 μM) did not affect γ power [103 ± 4% of baseline, *t*_(7)_ = 0.104, *P* = 0.920, Figure 7]. Further application of MA (20 μM) did not affect γ oscillations consistently; γ power was 120 ± 27% of triciribine [*t*_(7)_ = −0.612, *P* = 0.560, Figure 7]. These results show that Akt activation is required for MA-mediated enhancement of γ oscillations.

### The Effect of NMDA Receptor Antagonist on MA-Regulated γ Oscillations

**FIGURE 8:**
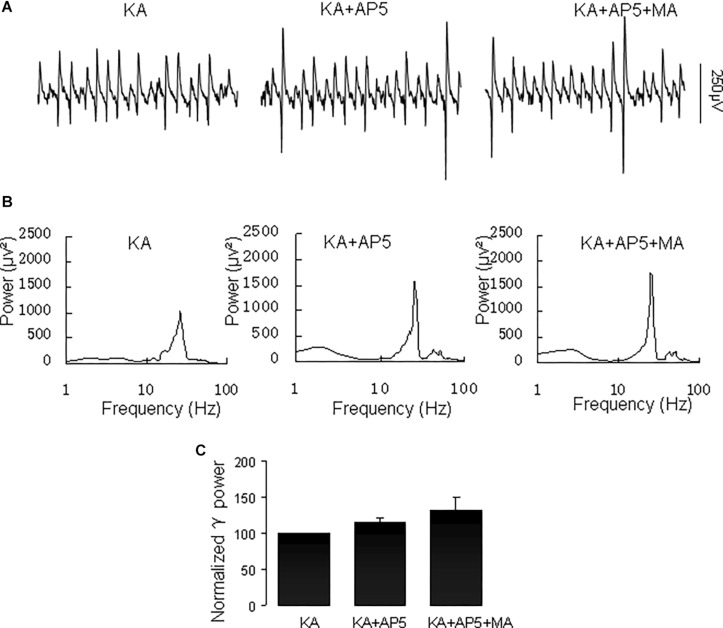
D-AP5 blocked the MA-induced increase in γ oscillations. **(A)** CA3 recordings for the presence KA alone, KA+ 10 μM D-AP5 and KA+10 μM D-AP5 + 20 μM MA. (B) The power spectra for the recordings in **A**. **(C)** γ power as percentage of preceding baseline for KA alone, KA+ D-AP5 and KA+ D-AP5 + MA.

The NMDA receptor plays a critical role in excitatory synaptic neurotransmission and plasticity, and NMDAR antagonists can cause hallucinations and increases in γ oscillations in hippocampus, nucleus accumbens and prefrontal cortex (Lee et al., 2017). We tested the effect of the NMDA receptor antagonist D-AP5 on the MA-mediated enhancement of γ oscillations. Application of D-AP5 (10 μM) alone slightly increased γ power (to 116 ± 5% of baseline, not different from the control *t*_(6)_ = −1.438, *P* = 0.200, Figure 8). Addition of MA (20 μM) had no consistent effect on γ oscillations [γ power was 111 ± 13% of D-APV, *t*_(6)_ = −1.065, *P* = 0.328, Figure 8]. These results indicate that NMDAR activation is necessary for MA-regulated γ enhancement.

## Discussion

Our results demonstrate that MA increases hippocampal γ oscillation strength *in vitro*, is dependent on both DR1 and DR2 activation, Akt activity and NMDAR activity, but not on ERK, PI3K or PKA activity.

### Signaling Pathways Involved in the MA-Mediated Enhancement of γ Oscillations

Methamphetamine increases dopamine concentrations in the tissue (Ares-Santos et al. (2013), probably through activation of trace amine-associated receptor 1, which inhibits uptake and promoting efflux by monoamine transporters (Miller, 2011). Both DR1 and DR2 antagonists blocked the effect of MA on γ power in this study, suggesting the involvement of both DR1 and DR2 in the MA-mediated γ oscillation enhancement. It is known that activation of the DR2-type DR4 increases hippocampal γ power (Andersson et al., 2012). Our results indicate DR1 plays a similar role as DR2 in the regulation of γ oscillations. This is in line with the observation that both selective DR1 and DR2 antagonists reverse MA-induced motor effects (Ujike et al., 1989). It is, however, surprising that the two DR antagonists have a similar effect on the MA-mediated modulation of γ oscillations, given that they have an opposite effect on cAMP-PKA signaling. It is possible that the opposing effect of two DRs on adenylyl cyclase may limit the PKA activity and the role of PKA on MA-mediated γ oscillations. In support of this notion, MA does not alter the expression of PKA catalytic subunit in hippocampus (Hayashi et al., 2010). Interestingly, both DR1 and DR2 agonists can increase phosphorylation levels of Akt (Brami-Cherrier et al., 2002).

The sensitivity to Akt inhibition reported here, suggests that MA enhancement of γ oscillations involves Akt signaling. This is in line with the observation that acute MA increases the Akt phosphorylation in cultured hippocampal neurons (Rau et al., 2011) and that MA-induced psychosis is associated with increased expression of Akt1, one of the three Akt isoforms (Nohesara et al., 2016). Phentermine, structurally similar to MA, induces conditional rewarding effects via activation of PI3K/Akt signaling in the NAc (Hong et al., 2016). However, in our experiments, PI3K inhibition did not affect the MA-modulation of γ oscillations. It is therefore possible that MA activates PI3K-independent Akt signaling (Brami-Cherrier et al., 2002).

Instead of PI3K, ERK may be an alternative upstream molecule of Akt (Brami-Cherrier et al., 2002). However, our results don’t support the involvement of ERK in MA-mediated enhancement of γ oscillations.

**FIGURE 9:**
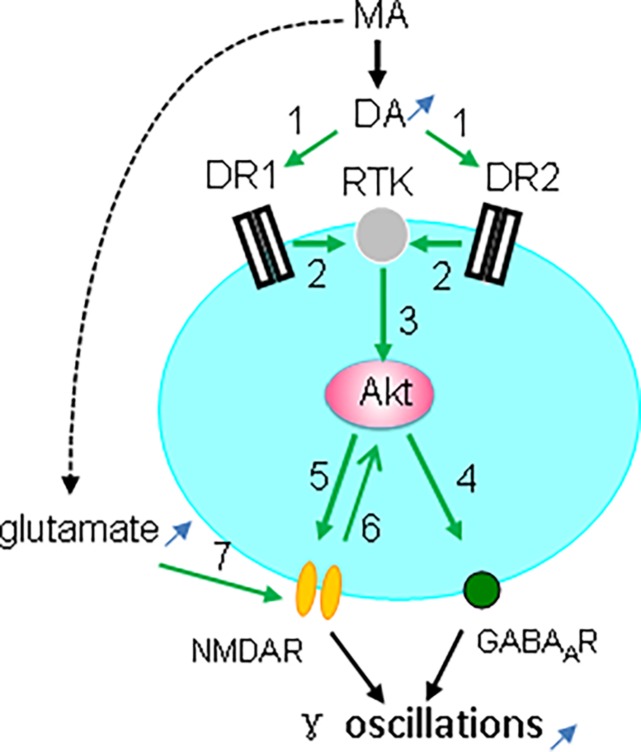
Diagram showing the possible mechanisms for MA-mediated enhancement ofγ oscillations. MA increases dopamine levels, which activates both DR1 and DR2 (green arrow 1, indicating activation), which causes transactivation of receptor tyrosine kinase (RTK) (2), and downstream kinase Akt phosphorylation (3). Akt activation increases postsynaptic GABA_A_ receptors (4) and activates NMDAR (5), which may also activate Akt (6). In addition MA can increase glutamate levels that activate NMDA receptors (7). NMDAR activation and the increased GABA_A_R expression can both contribute to the enhancement of γ oscillations.

Taken together, our observations indicate that the MA-mediated enhancement of γ oscillations acts through DR1/DR2-mediated Akt activation (Figure 9). Our results are in agreement with previous reports (Mannoury la Cour et al., 2011; Swift et al., 2011; Chen et al., 2012) but not with DR2-mediated Akt inhibition (Beaulieu et al., 2007b). The exact mechanism for DR-induced activation of Akt remains therefore to be further investigated. One of the possibilities is that DR1 or DR2 activates Akt via receptor tyrosine kinase (RTK) transactivation (Beaulieu et al., 2011), as it has been shown that stimulation of DR1 or DR2 causes activation of the epidermal growth factor receptor or TrkB RTK (Swift et al., 2011).

In addition to dopamine, MA also increases tissue levels of norepinephrine and serotonin. However, if anything, norepinephrine suppresses kainate-induced oscillations *in vitro* (Wojtowicz et al., 2009) and norepinephrine released from the locus coeruleus inhibits gamma *in vivo* (Walling et al., 2011). This may be explained by the suppression of excitatory synaptic transmission in hippocampus by norepinephrine (Scanziani et al., 1993). Serotonin also suppresses kainate-induced oscillations (Wojtowicz et al., 2009; Johnston et al., 2014). Thus, although we have not tested the role of norepinephrine or serotonin in current study, neither NE nor serotonin are likely to contribute to the MA-mediated enhancement of hippocampal γ oscillations.

### Role of NMDAR on the MA-Mediated Enhancement of γ Oscillations

The dependence on NMDAR activity of the MA-mediated enhancement of γ oscillations in our experiments, suggest a crucial role for NMDAR-linked signaling. This is in agreement with the MA-induced enhancement of NMDA currents in mesencephalic dopamine neurons (Li et al., 2017) and with the MA-induced increase in NR1 expression in the striatum (Kerdsan et al., 2009). We previously demonstrated the involvement of NMDAR in γ enhancement mediated by low concentrations of the stimulant nicotine (Wang et al., 2015).

Interestingly, Akt activity is dynamically regulated by synaptic activity that is coupled to NMDA receptor activation (Sutton and Chandler, 2002). Akt-mediated NMDAR activation is via phosphorylation of the NMDAR subunit NR2C (Sanchez-Perez et al., 2006; Chen and Roche, 2009). The MA-mediated enhancement of γ oscillations is therefore likely due to Akt-mediated activation of NMDARs (Figure 9). In addition, MA may activate NMDARs by increasing extracellular glutamate concentration via neuronal excitatory amino acid transporter 3 internalization (Li et al., 2017).

### Possible Mechanism Underlying the MA-Mediated Enhancement of γ Oscillations

CA3 γ oscillations emerge from the interplay between fast-firing parvalbumin-expressing interneurons and pyramidal cells (Mann and Paulsen, 2005; Bartos et al., 2007). Activation of Akt may enhance γ oscillations through an increase in postsynaptic GABA_A_ receptor density and GABAergic synaptic transmission (Wang et al., 2003; Chen et al., 2014; Figure 9). Alternatively, modulation of NMDAR activity may affect γ oscillations, because parvalbumin-expressing interneurons, express NMDARs, which provide tonic depolarization and thus drive γ oscillations (Carlen et al., 2012; Figure 9).

### Functional Implications of the MA-Mediated Enhancement of γ Oscillations

Although our observations are based on the isolated γ oscillation-generating CA3 network in the *in vitro* kainate model, it is likely that similar MA-mediated enhancement γ oscillations will take place in vivo, where modulation by midbrain and brainstem inputs adds a level of complexity.

The MA-induced enhancement of γ oscillations is in line with the observation that MA treatment increases γ oscillations in the NAc of freely moving rats (Morra et al., 2012) and that amphetamine or the dopamine agonist apomorphine increases γ power in the rat neocortex (Pinault, 2008). These studies suggest that MA can enhance γ oscillations in multiple mutually connected brain areas. γ oscillations have been suggested to synchronize the activity of neural ensembles and to control the information flow within and between anatomically connected networks (Konig et al., 1995; Womelsdorf et al., 2007; Colgin, 2011; Buzsaki and Wang, 2012; Akam and Kullmann, 2014; Fries, 2015). These studies suggest that MA can enhance γ oscillations in functionally connected brain areas involved in psychosis (Ma and Leung, 2010; Lee et al., 2017), which may cause aberrantly strong connectivity between these areas, contributing to MA-induced psychosis in normal subjects (Wearne and Cornish, 2018). In subjects with low levels of attention, the MA-mediated enhancement of γ oscillations may restore functional connectivity and increase attention, which may explain the use of MA in the treatment of attention-deficit hyperactivity disorder in children (Hart et al., 2012).

In addition to dopamine, MA increases norepinephrine and serotonin levels (Rothman et al., 2001), which may contribute to MA-induced psychosis in humans and psychosis-like repetitive behaviors in animals (Morra et al., 2010).

It is worth to mention that the enhancement of γ oscillations by acute MA is mechanistically different from the effect of other psychostimulants such as ketamine, which also increases cortical γ power and causes cognitive deficits and psychosis (Furth et al., 2017). In contrast to ketamine, acute MA enhancement of γ power is associated with NMDAR activation. This difference may explain why ketamine is associated with impaired cognitive function, whereas acute MA is associated with improved spatial memory in human addicts (Mahoney et al., 2011, Rau et al., 2012) and enhanced synaptic plasticity in rodents (Hori et al., 2010).

## Author Contributions

YL, XX, HX, YW, and YJ conducted the experiments. YL, XX, XY, JW, and YZ analyzed the data. CL and RZ designed and directed the study. CL, YL, and MV wrote the manuscript. MV and CL revised the manuscript.

## Conflict of Interest Statement

The authors declare that the research was conducted in the absence of any commercial or financial relationships that could be construed as a potential conflict of interest.
